# Surfactant therapy for acute respiratory failure in children: a systematic review and meta-analysis

**DOI:** 10.1186/cc5944

**Published:** 2007-06-15

**Authors:** Mark Duffett, Karen Choong, Vivian Ng, Adrienne Randolph, Deborah J Cook

**Affiliations:** 1 Department of Critical Care, McMaster Children's Hospital, 1200 Main St. W., Hamilton, Ontario L8S 4J9, Canada; 2 Grand River Hospital, 835 King St. West, Kitchener, Ontario N2G 1G3, Canada; 3 Children's Hospital Boston, 300 Longwood Avenue, MSICU, FA-108, Boston, MA 02115, USA; 4 Department of Clinical Epidemiology and Statistics, McMaster University, 1200 Main St. W., Hamilton, Ontario L8N 3Z5, Canada

## Abstract

**Introduction:**

Exogenous surfactant is used to treat acute respiratory failure in children, although the benefits and harms in this setting are not clear. The objective of the present systematic review is to assess the effect of exogenous pulmonary surfactant on all-cause mortality in children mechanically ventilated for acute respiratory failure.

**Methods:**

We searched the MEDLINE, EMBASE, CINAHL and Ovid Healthstar databases, the bibliographies of included trials and review articles, conference proceedings and trial registries. We included prospective, randomized, controlled trials of pulmonary surfactant that enrolled intubated and mechanically ventilated children with acute respiratory failure. We excluded trials that exclusively enrolled neonates or patients with asthma. Two reviewers independently rated trials for inclusion, extracted data and assessed the methodologic quality. We quantitatively pooled the results of trials, where suitable, using a random effects model.

**Results:**

Six trials randomizing 314 patients were included. Surfactant use reduced mortality (relative risk = 0.7, 95% confidence interval = 0.4 to 0.97, *P *= 0.04), was associated with increased ventilator-free days (weighted mean difference = 2.5 days, 95% confidence interval = 0.3 to 4.6 days, *P *= 0.02) and reduced the duration of ventilation (weighted mean difference = 2.3 days, 95% confidence interval = 0.1 to 4.4 days, *P *= 0.04).

**Conclusion:**

Surfactant use decreased mortality, was associated with more ventilator-free days and reduced the duration of ventilation. No serious adverse events were reported.

## Introduction

Acute respiratory failure remains the primary indication for admission to North American paediatric intensive care units (PICUs) and accounts for significant mortality, morbidity and resource utilization [1]. Respiratory infections, in particular pneumonia and severe bronchiolitis, are the most common causes of respiratory failure requiring mechanical ventilation in children [1].

Alterations in endogenous surfactant play a role in the pathogenesis of many causes of acute lung injury (ALI) and acute respiratory distress syndrome (ARDS) [2]. Surfactant dysfunction, destruction and inactivation have also been demonstrated in children with acute respiratory insufficiency due to bronchiolitis [3,4]. The administration of exogenous surfactant may reduce the need for mechanical ventilation and its associated sequelae by restoring surfactant levels and function. Inspired by the success of surfactants in reducing mortality and the need for mechanical ventilation in neonatal respiratory distress syndrome [5], investigators have studied exogenous surfactant in other populations with various causes of respiratory failure. Trials of surfactant in adults with ALI and ARDS have not demonstrated a mortality benefit [6-9], perhaps due to inherent differences in the aetiology of lung injury in adults, the design features of the trials, the mode and timing of surfactant administration or the type and dose of surfactant used. In children with respiratory failure, the efficacy of exogenous surfactant has been suggested in uncontrolled studies [10,11]. The relatively low mortality rate, the diversity of the study populations and the shorter duration of mechanical ventilation are factors that make large-scale randomized controlled trials in this population challenging to conduct. Two of the largest trials were stopped early due to slower than expected enrolment [12,13]. While the use of surfactant in ARDS/ALI has not been previously systematically reviewed, its use in children with bronchiolitis has been [14].

We anticipated that including trials enrolling children with acute respiratory failure from a variety of causes would result in a heterogeneous population and would increase the generalizability of the results. Our confidence in the results of the present review would also be increased if a consistent effect is shown in subgroups and across a spectrum of disease severity.

The primary objective of the systematic review is to assess the effect of the administration of pulmonary surfactant compared with no therapy or with placebo on all-cause mortality (at or before hospital discharge) in mechanically ventilated children with acute respiratory failure.

## Methods

### Trial selection

We included trials that were prospective, that were randomized, that enrolled children intubated and mechanically ventilated for acute respiratory failure and that compared the intratracheal administration or nebulization of at least one dose of natural or artificial pulmonary surfactant with a placebo or no intervention. We excluded trials exclusively enrolling neonates or patients with asthma. We used the trial authors' definitions of paediatric.

### Outcome measures

The primary outcome measure was all-cause mortality at or before hospital discharge. Secondary outcomes were ventilator-free days to day 28 (a composite of mortality and duration of ventilation, defined as days alive and free from mechanical ventilation) [15], the duration of mechanical ventilation (from intubation to extubation, death or trial withdrawal), the duration of PICU stay, the use of rescue therapy (such as extracorporeal membrane oxygenation, high-frequency oscillatory ventilation, open label surfactant and nitric oxide), and complications and adverse effects as reported by the trial authors.

### Searching

One of us searched for published and unpublished trials, examining trial registries, conference proceedings and the bibliographies of any identified trials and relevant reviews (the search strategy is available upon request). We polled paediatric intensivists and pharmacists at our institution for additional trials. We selected search terms from the keywords and MESH terms of previous surfactant trials and from the generic and brand names of commercially available surfactants. We imposed no language restrictions.

### Trial selection

One of us screened the title (and abstract if required) of all citations retrieved. We selected citations for further evaluation if they reported the administration of at least one dose of surfactant to at least one child or if the title or abstract did not give enough information to make an assessment. Two reviewers independently reviewed all citations meeting criteria for further review and applied the inclusion criteria. Disagreements between reviewers were resolved by consensus in consultation with a third reviewer. We considered agreement between reviewers to be acceptable if the kappa value was greater than 0.8.

### Quality assessment

We used the following characteristics to assess the methodologic quality: allocation concealment (sealed envelopes or central randomization were considered adequate), blinding (which of the trial personnel and caregivers were blinded, and the methods used to ensure blinding), completeness of follow-up (assessed by the number of patients randomized for whom there were no outcomes), similarity of the groups at baseline (with respect to known prognostic factors: age, aetiology, severity of illness as measured by the Pediatric Risk of Mortality score, and immunosuppression), whether a standard or recommended strategy for mechanical ventilation was used, and whether *a priori *criteria for the use of co-interventions were used. Effective blinding of surfactant is challenging because of the large volumes of milky fluid administered, which can often be seen by caregivers in the patients' ventilator tubing or endotracheal tube, particularly during suctioning.

We pretested and refined the developed forms on two trials of surfactant therapy for adults, and clarified definitions based on feedback from the reviewers. Two reviewers then independently used these forms to abstract trial quality, blinded to the authors, the journal, the country of origin and the results. We resolved any disagreements by consensus in consultation with a third reviewer if needed.

### Data abstraction

After pretesting and refining the forms on two trials of surfactant therapy in adults and clarifying definitions based on feedback from the reviewers, two reviewers then independently abstracted the data. Reviewers were only provided with a full-text version of the trials from which the introduction, conclusions and discussion were omitted and from which the author, journal and country of origin were deleted. We thereafter examined these sections of the reports for any missing data. We resolved any disagreements between reviewers by consensus in consultation with a third reviewer if needed. We asked the authors to supply data not included in the published reports. Two reviewers performed data entry in duplicate.

### Statistical methods

We quantitatively pooled the results of individual trials when possible. We expressed the treatment effect as a relative risk for dichotomous outcomes and as a weighted mean difference for continuous outcomes with 95% confidence intervals. We considered effects statistically significant if *P *< 0.05. A *z *test was used to statistically test the estimates of treatment effect between groups [16]. We assessed heterogeneity among trials using the *I*^2 ^statistic, and considered an *I*^2 ^value greater than 50% to indicate substantial heterogeneity [17]. RevMan 4.2 software and a random effects model were used to perform the analyses [18]. We chose the random effects model because it gives a more conservative estimate of the precision of the treatment effects and because the true effect of the intervention probably varies given the different populations enrolled in these trials [19]. A subgroup analysis was planned based on the aetiology of respiratory failure (trials enrolling exclusively patients with respiratory syncytial virus (RSV)/severe bronchiolitis compared with all other trials) if sufficient data were available, because these trials were likely to enrol a younger, more homogeneous, population with a lower predicted risk of mortality. We also planned sensitivity analysis based on methodological features of the included trials (trials reporting adequate allocation concealment compared with all other trials).

## Results

### Trial flow

**Figure 1:**
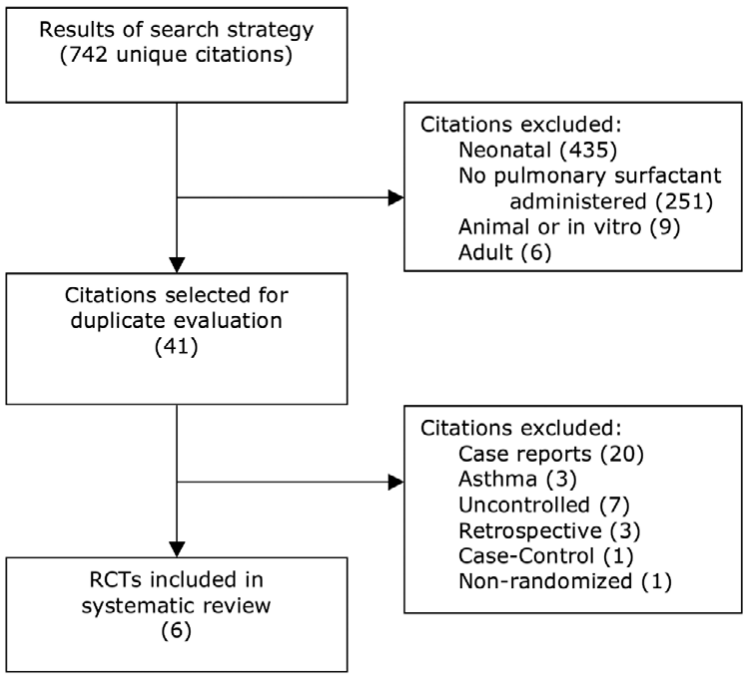
Flow diagram of included trials. RCTs, randomized controlled trials.

We identified 742 unique citations, six of which met our inclusion criteria (Figure 1 outlines the reasons for exclusion). Most reports excluded enrolled neonates or were retrospective or uncontrolled in design. Chance corrected agreement was excellent (kappa = 0.91, 95% confidence interval = 0.73–1.1).

### Methodologic quality of included trials

Table 1 presents a complete description of our quality assessment. Only one trial did not report allocation concealment [20]. Although effective blinding of surfactant is challenging, two trials reported blinding of the PICU team [12,20]. The two groups were generally well matched in terms of baseline characteristics in most trials. The most significant imbalance was the numerically higher number of immunosuppressed patients in the placebo group. These patients had higher mortality (56%) than the immunocompetent group (13%). The authors attempted to adjust for this imbalance with logistic regression, which suggested that the treatment effect seemed to be relatively consistent between the two groups [12]. Only one trial reported *a priori *criteria for rescue therapy [13].

**Table 1:** Trial methodological quality

Trial	Allocation concealment reported? (Method used)	Who was reported to be blinded? (Who administered the intervention?)	Completeness of follow-up reported?^a^	Groups similar at baseline?^b^	Ventilation algorithm described?	Criteria for rescue therapy^c^
Luchetti, 1998	Yes (sealed envelopes)	Not reported	Not reported	Yes	Yes	Not reported
Willson, 1999	Yes (opaque sealed envelopes)	Unblinded	91%	Lower PRISM scores in control group (9 versus 12)	Yes	No
Tibby, 2000	Not reported	Care providers (investigators not involved in patient care)	Not reported	Yes	Yes	Not reported
Luchetti, 2002	Yes (sealed envelope)	Outcome assessors (investigators)	Not reported	Yes	Yes	No
Moller, 2003	Yes (central telephone)	Unblinded	87%	Yes	Yes	Yes
Willson, 2005	Yes (opaque sealed envelopes)	Care providers, investigators (respiratory therapist not involved in patient care)	100%	More immunosuppressed in control group (30 versus 22)	Yes	No

^a^Patients randomized but not included in the analysis. ^b^Potentially clinically significant differences in age, aetiology, severity of illness (Pediatric Risk of Mortality (PRISM) score) and immunosuppression. ^c^*A priori *criteria for the use of extracorporeal membrane oxygenation, high-frequency oscillatory ventilation, nitric oxide and open-label surfactant.

### Description of included trials

Table 2 describes the included trials. Three trials enrolled exclusively infants with RSV-induced respiratory failure [20,21] or with severe bronchiolitis [22]. The remaining three trials enrolled a heterogeneous group of patients with ARDS or ALI [12,23,24]. While the individual treatment protocols varied, all trials used comparable doses (50–100 mg/kg phospholipids) of natural or modified natural surfactants and each patient typically received one or two doses. A variety of interventions were used in the control groups: no intervention, air placebo or similar sedation and ventilation manoeuvres without a placebo. Although one study [20] used a modified natural surfactant, all the products used contained surfactant proteins B and C. All studies administered surfactant early in the course of respiratory failure; most patients were treated within 12–48 hours of requiring mechanical ventilation.

**Table 2:** Summary of trial design

Trial	Patient population^a^	Number of centres	Surfactant (dose^b^)	Control	Primary outcome	Duration of follow-up	Funding source
Luchetti, 1998	Severe bronchiolitis	Unclear, probably 1	Poractant – porcine (50 mg/kg single dose)	None	Unclear	PICU discharge	Not reported
Willson, 1999	ARDS/ALI	8	Calfactant – bovine (2,800 mg/m^2 ^every 12 hours for 1–4 doses)	None	Mortality	Hospital discharge	Partial support by surfactant manufacturer
Tibby, 2000	RSV-induced respiratory failure	Unclear, probably 1	Beractant – modified bovine (100 mg/kg every 24 hours for 2 doses)	Air placebo	Indices of gas exchange	Hospital discharge	Not reported
Luchetti, 2002	RSV-induced respiratory failure	6	Poractant – porcine (50 mg/kg dose every 24 hours for 1–2 doses)	Sedation and manual ventilation	Vent days and PICU stay	PICU discharge	Not reported
Moller, 2003	ARDS	19	Bovactant – bovine (100 mg/kg, 1–2 doses within 48 hours)	None	Change in PaO_2_/FiO_2 _at 48 hours	30 days	Surfactant manufacturer
Willson, 2005	ARDS/ALI	21	Calfactant – bovine (2,800 mg/m^2 ^– if <10 kg, 105 mg/kg – every 12 hours for 1–2 doses)	Air placebo	Ventilator-free days at day 28	Hospital discharge	Surfactant manufacturer

ALI, acute lung injury; ARDS, acute respiratory distress syndrome; PICU, paediatric intensive care unit; RSV, respiratory syncytial virus. ^a^See Additional file 1 for the complete inclusion criteria and exclusion criteria. ^b^Dose expressed as mg/kg phospholipids.

The baseline characteristics of the patients are presented in Table 3. While there was significant heterogeneity among and within trials with respect to age and cause of respiratory failure, we considered the initial Pediatric Risk of Mortality scores and the initial PaO_2_/FiO_2 _ratios to be clinically comparable.

**Table 3:** Baseline characteristics

Trial	Aetiology of respiratory failure^a^		Treatment group	Age (years)	Initial PaO_2_/FiO_2_	PRISM score
Luchetti, 1998	Bronchiolitis Pneumonia	100%	Surfactant	0.87 (0.15)	118 (15.8)	Not reported
		50%	Placebo	0.93 (0.17)	122 (12.0)	Not reported
Willson, 1999	ARDS	31%				
	Pneumonia	26%	Surfactant	5.0 (5.34)	102 (53)	12 (6)
	RSV	17%	Placebo	4.5 (5.66)	105 (42)	9 (4)
	Near-drowning	7%				
	Sepsis	7%				
	Other	14%				
Tibby, 2000	RSV	100%	Surfactant	0.17 (0.14, 0.27)^b^	146 (134, 171)^b^	11 (11, 15)^b^
			Placebo	0.13 (0.09, 0.18)^b^	160 (106, 205)^b^	13 (11, 15)^b^
Luchetti, 2002	RSV	100%	Surfactant	0.73 (0.675)	Not reported	12 (1.1)
	Pneumonia	45%	Placebo	0.62 (0.542)	Not reported	12 (1.2)
Moller, 2003	Pneumonia	68%	Surfactant	3.5 (range 0–13)	71 (13.7)	12 (6.5)
	Sepsis	32%	Placebo	4.5 (range (0–12)	64 (16.2)	11 (4.5)
Willson, 2005	ARDS or sepsis	35%	Surfactant	7.2 (6.4)	128 (54)	15 (9.4)
	Pneumonia	42%	Placebo	6.7 (6.4)	126 (73)	14 (7.9)
	RSV	7%				
	Near-drowning	5%				
	Other	11%				

Values expressed as the mean (standard deviation) unless otherwise indicated. ARDS: acute respiratory distress syndrome; PRISM, Pediatric Risk of Mortality; RSV: respiratory syncytial virus. ^a^Totals may be greater than 100% due to rounding and because multiple aetiologies were reported for some patients. ^b^Median (interquartile range).

### Primary outcome: mortality

**Figure 2:**
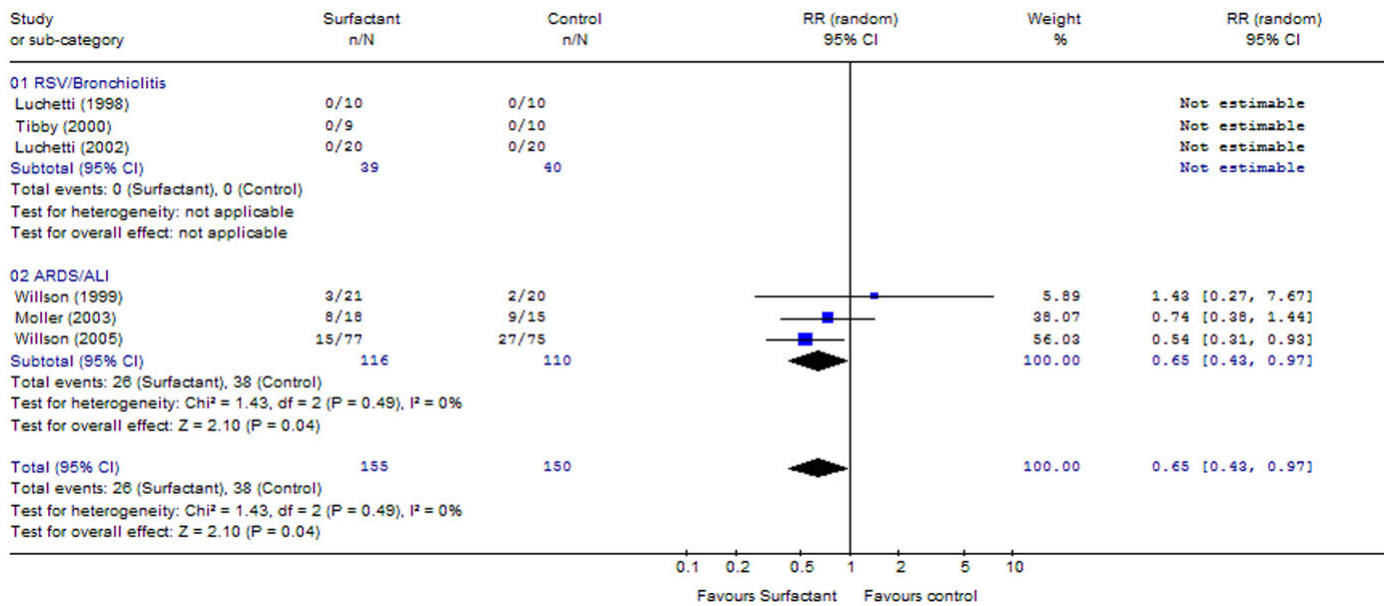
Meta-analysis of trials of surfactant in children with acute respiratory failure: Mortality. ALI, acute lung injury; ARDS, acute respiratory distress syndrome; 95% CI, 95% confidence interval; RR, relative risk; RSV, respiratory syncytial virus.

Mortality data were available for all six trials, randomizing 311 patients and reporting data for 305 patients. There were no deaths reported in the three RSV/severe bronchiolitis trials; thus our estimate is based on three trials randomizing 232 patients, 64 of whom died. In the pooled analysis, surfactant was associated with significantly lower mortality (relative risk = 0.7, 95% confidence interval = 0.4–0.97, *P *= 0.04). There was no evidence of heterogeneity (*I*^2 ^= 0%) (Figure 2).

### Secondary outcomes

#### Ventilator-free days to day 28

**Figure 3:**
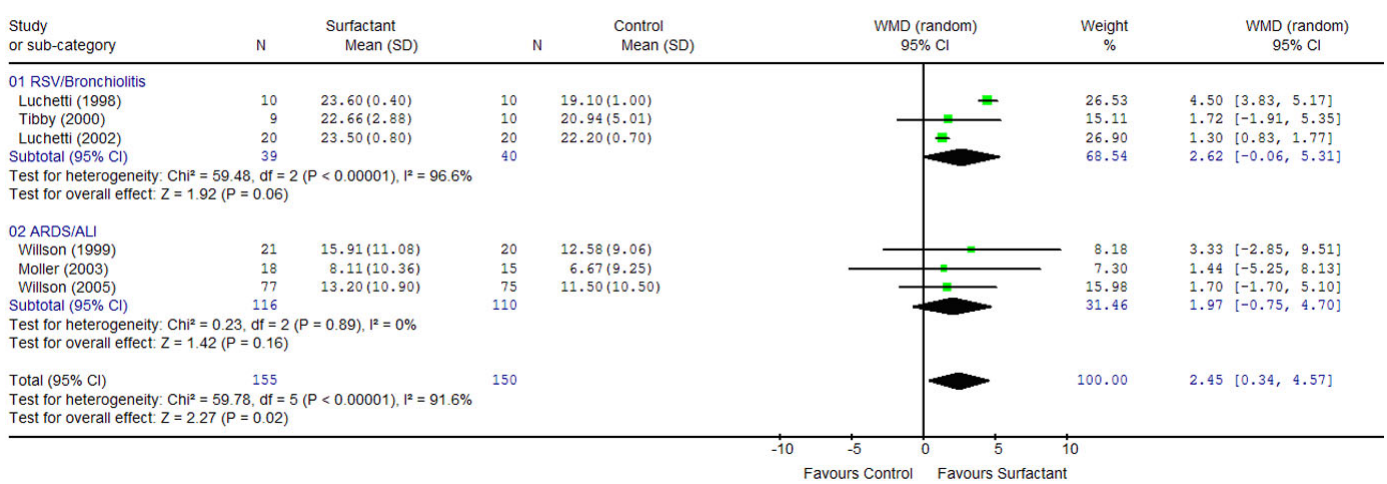
Meta-analysis of trials of surfactant in children with acute respiratory failure: Ventilator-free days. ALI, acute lung injury; ARDS, acute respiratory distress syndrome; 95% CI, 95% confidence interval; RSV, respiratory syncytial virus; SD, standard deviation; WMD, weighted mean difference.

The number of ventilator-free days to day 28 was available for six trials randomizing 311 patients and reporting data for 305 patients. In the pooled analysis, surfactant was associated with significantly more ventilator-free days (weighted mean difference = 2.5 days, 95% confidence interval = 0.3–4.6 days, *P *= 0.02) (Figure 3).

#### Duration of mechanical ventilation

**Figure 4:**
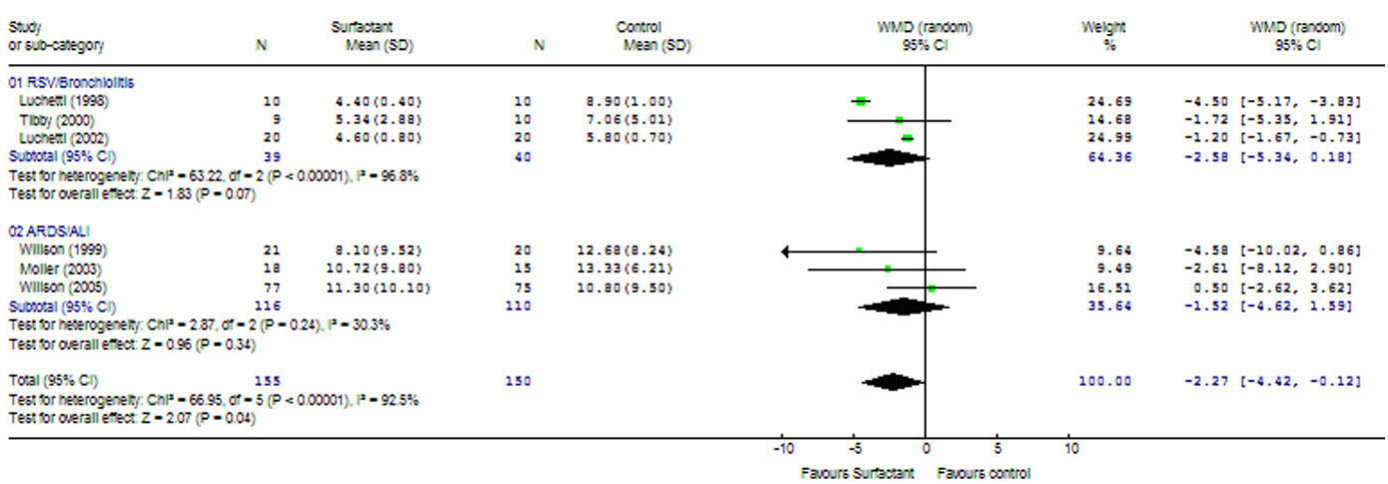
Meta-analysis of trials of surfactant in children with acute respiratory failure: Duration of mechanical ventilation. ALI, acute lung injury; ARDS, acute respiratory distress syndrome; 95% CI, 95% confidence interval; RSV, respiratory syncytial virus; SD, standard deviation; WMD, weighted mean difference.

The duration of mechanical ventilation was available for six trials randomizing 311 patients and reporting data for 305 patients. In the pooled analysis, surfactant was associated with a significantly shorter duration of mechanical ventilation (weighted mean difference = 2.3 days, 95% confidence interval = 0.1–4.4 days, *P *= 0.04) (Figure 4).

#### Duration of PICU stay

**Figure 5:**
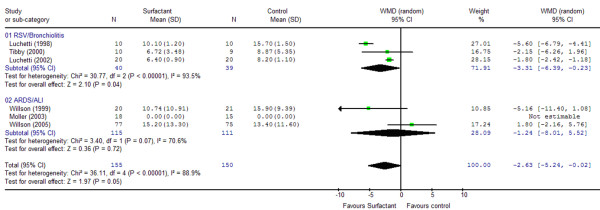
Meta-analysis of trials of surfactant in children with acute respiratory failure: Duration of PICU stay. ALI, acute lung injury; ARDS, acute respiratory distress syndrome; 95% CI, 95% confidence interval; PICU, paediatric intensive care unit; RSV, respiratory syncytial virus; SD, standard deviation; WMD, weighted mean difference.

The duration of PICU stay was available for five trials randomizing 273 patients and reporting data for 272 patients. In the pooled analysis, surfactant was associated with a shortened duration of PICU stay (weighted mean difference = 2.6 days, 95% confidence interval = 0.02–5.2 days, *P *= 0.05), but this difference was not statistically significant (Figure 5).

#### Use of rescue therapy

Data on the use of rescue therapy were available for six trials randomizing 311 patients and reporting data for 305 patients. In the pooled analysis, the surfactant was associated with a significantly lower use of rescue therapy (relative risk = 0.4, 95% confidence interval = 0.3–0.7, *P *< 0.0001). There was no evidence of heterogeneity (*I*^2 ^= 0%). This summary estimate should be interpreted with caution as only one trial reported a protocol for initiating rescue therapy. The decision to use a rescue therapy, particularly an open-label surfactant, may be influenced by knowledge of the patient's allocation; furthermore, only two trials reported blinded caregivers and the methods used to ensure blinding may not be adequate.

#### Adverse events

Surfactant therapy was well tolerated (see Table 4), but only three of the trials reported any definitions or *a priori *criteria or of collecting adverse events [12,21,23]. Transient hypotension and transient hypoxia were the most commonly reported adverse events in the largest trial. These responded to a brief adjustment in ventilation, to a slowing of the rate of surfactant administration or to fluid administration. There was no difference in the incidence of air leaks in the two trials that reported this outcome. No patient was withdrawn from any of the trials because of adverse events. We did not pool the data on adverse events associated with the trial interventions from the six trials because of the inconsistent manner in which the events were documented and reported.

**Table 4:** Reported adverse events

Trial	Adverse events reported	
	
	Surfactant (*n *= 157)	Control (*n *= 150)
Luchetti, 1998	'No adverse haemodynamic effects were noted'	'No complications were reported in either group'
	'No complications were reported in either group'	
Willson, 1999	One with transient bronchospam	One with air leak
	Two with air leaks	One with air leak and pulmonary interstitial emphysema
	One with pulmonary interstitial emphysema	One plugged endotracheal tube
Tibby, 2000	'No complications were noted after surfactant administration'	Not reported
Luchetti, 2002	'No complications due to the treatment were observed in the surfactant-treated group'	'... no complications were found in the control group'
Moller, 2003	'No treatment associated adverse events were observed in the surfactant group; however, the expected risk of intermittent obstruction of the endotracheal tube with a short time deterioration on oxygenation was observed in 3 patients'	Not reported
Willson, 2005	Hypotension 9%	Hypotension 1%
	Transient hypoxia 12%	Transient hypoxia 3%
	Air leaks 13%	Air leaks 16%
	Nosocomial pneumonia 6%	Nosocomial pneumonia 11%

### Subgroup analysis

The effect of surfactant on ventilator-free days, the duration of mechanical ventilation and the duration of PICU stay was not significantly different when we compared the three trials that enrolled exclusively patients with RSV/severe bronchiolitis with the three other trials (Table 5). A 100% survival in the bronchiolitis trials subgroup precludes formal subgroup analysis for the primary outcome of mortality.

**Table 5:** Subgroup analysis

Outcome	Bronchiolitis trials	All other trials	*P *value
Mortality	Unable to assess		
Ventilator-free days			
Weighted mean difference (days)	2.6	2.0	
95% confidence interval	-0.1 to 5.3	-0.8 to 4.7	0.76
Duration of mechanical ventilation			
Weighted mean difference (days)	2.6	1.5	
95% confidence interval	-0.1 to 5.3	-1.6 to 4.6	0.60
Duration of paediatric intensive care unit stay			
Weighted mean difference (days)	3.3	1.2	
95% confidence interval	0.2 to 6.4	-5.5 to 8.0	0.58

### Sensitivity analysis

All but one of the included trials reported adequate allocation concealment (defined as sealed envelopes or central telephone randomization). Since there were no deaths in this trial we could not assess the effect of inadequate allocation concealment on mortality. Pooling the five remaining trials did not change the direction of the effect and did not significantly change the point estimates for the secondary outcomes of ventilator-free days, duration of ventilation or duration of PICU stay (Table 6).

**Table 6:** Sensitivity analysis

Outcome	Trials reporting adequate allocation concealment	All other trials	*P *value
Mortality	Unable to assess		
Ventilator-free days			0.68
Weighted mean difference (days)	2.6	1.7	
95% confidence interval	0.3 to 4.7	-1.9 to 5.4	
Duration of mechanical ventilation			0.76
Weighted mean difference (days)	2.4	1.7	
95% confidence interval	-0.01 to 4.7	-1.9 to 5.4	
Duration of paediatric intensive care unit stay			0.85
Weighted mean difference (days)	2.7	2.2	
95% confidence interval	-0.3 to 5.7	-2.0 to 6.3	

## Discussion

In the present systematic review and meta-analysis of the effect of surfactant for critically ill children with acute respiratory failure we found that surfactant therapy significantly reduced our primary outcome of mortality. Surfactant was associated with more ventilator-free days, with decreased duration of ventilation and with less use of rescue therapy as compared with standard therapy. There was no significant difference in the duration of PICU stay. Surfactant therapy was well tolerated; while transient hypoxia and hypotension were reported during surfactant administration, no study reported any serious adverse events. The patients enrolled in these trials are representative of the heterogeneous group of children with early, severe acute respiratory failure that is seen in clinical practice. These patients had similar severity of illness scores and a similar degree of respiratory failure (as measured by Pediatric Risk of Mortality scores and PaO_2_:FiO_2 _ratios).

The heterogeneity of results for our primary outcome of mortality was low. The presence of significant heterogeneity reduces the strength of inferences we can make regarding the effect of surfactant on the secondary outcomes of ventilator-free days, duration of ventilation and duration of PICU stay. Separately pooling the trials that exclusively enrolled patients with RSV/severe bronchiolitis and those enrolling patients with ARDS/ALI from a variety of causes did not significantly reduce the heterogeneity. Changing ventilation strategies and the use of a variety of natural and modified natural surfactants may have increased the heterogeneity of our results. Ventilation strategies, such as the use of lower tidal volumes and earlier use of high-frequency oscillatory ventilation, have evolved significantly in the 10-year span over which the included trials were conducted [25-27]. The surfactants used in the included trials were all natural or modified natural surfactants; however, these surfactants may have slightly different effects on oxygenation and compliance due to the differences in phospholipid and surfactant protein composition, which may have influenced individual study results.

The strengths of the present review include a comprehensive search strategy, broad inclusion criteria (resulting in a representative, heterogeneous population) and abstraction of clinically important outcomes in duplicate, independently blinded to information that may bias evaluation. The strength of the inference we can make from our subgroup analysis is limited because we were unable to extract all subgroup data from these trials. Access to individual patient data would allow better examination of the treatment effect in subgroups of patients and would facilitate further exploration of possible causes of heterogeneity.

We found that mortality was very different between the trials that exclusively enrolled patients with RSV/severe bronchiolitis and those that enrolled patients with ARDS/ALI from a variety of causes. We pooled the results because both conditions result in abnormal surfactant function and because of the substantial overlap between the two groups; up to 17% of children in the ARDS/ALI trials had RSV and up to 50% of the children in some bronchiolitis studies also had pneumonia.

The reduction in mortality and the increased ventilator-free days have important implications as very few trials in paediatric critical care suggest a favourable impact on mortality [28]. The present review suggests that surfactant could be an important adjunct in the management of paediatric respiratory failure. Uncertainty exists, however, about the reproducibility of treatment effects generated from relatively small unblinded trials; questions remain about adverse affects, which may be undetected or under-reported in this literature. Also, a large proportion of patients and events are reported in one trial [12]. Furthermore, issues of the optimal dose and the timing of administration, and which patients are most likely to derive benefit, should be studied in further adequately powered multicentre trials. The Pediatric Acute Lung Injury and Sepsis Investigators network is planning a large rigorous randomized trial enrolling children with acute hypoxemic respiratory failure to address these issues.

## Conclusion

Surfactant use decreased mortality, was associated with more ventilator-free days and reduced the duration of ventilation. No serious adverse events were reported. Most trials enrolled small numbers of children, and further well-designed and adequately powered multicentre trials are therefore required.

## Key messages

• Surfactant decreased mortality in a heterogeneous population of children with acute respiratory failure.

• Surfactant was associated with more ventilator-free days and a reduced duration of ventilation.

• No serious adverse events were reported.

• Further well-designed and adequately powered multicentre trials are required.

## Abbreviations

ALI = acute lung injury; ARDS = acute respiratory distress syndrome; FiO_2 _= fractional inspired oxygen; PaO_2 _= arterial oxygen tension; PICU = paediatric intensive care unit; RSV = respiratory syncytial virus.

## Competing interests

The authors declare that they have no competing interests.

## Authors' contributions

MD conceived of this review. MD, KC, VN and DJC participated in the design. MD and VN extracted data and assessed the quality of the included studies. MD, KC, DJC and AR helped to draft the manuscript. All authors read and approved the final manuscript.

## Supplementary Material

Additional file 1 A Word file containing a table listing individual trial inclusion criteria and exclusion criteria. Click here for file
