# Supplementary material for: Surfactant therapy for acute respiratory failure in children: a systematic review and meta-analysis
Source: Crit Care. 2007 Jun 15;11(3):R66. doi: 10.1186/cc5944 (PMC2206432; doi:10.1186/cc5944)
Supplement: Additional file 1 — A Word file containing a table listing individual trial inclusion criteria and exclusion criteria. [file cc5944-S1.doc]

| **Trial** | Inclusion criteria | **Exclusion criteria** |
| --- | --- | --- |
| Luchetti  1998 | severe bronchiolitis , vented for 24 h without significant improvement  PaO_2_/FiO_2_ <21 kpa and FiO_2_>60 on admission and at 24h  PaCO_2_>5.6 kpa  PIP >35 | not reported |
| Willson  1999 | clinical and radiographic evidence of diffuse parenchymal lung injury  less than 24 hours mechanical vented  oxygenation index >7  term infants >1 day to 18 yrs | chronic lung disease  status asthmaticus  cardiogenic pulmonary edema or congestive heart failure or uncorrected congenital heart disease  lethal brain injury |
| Tibby  2000 | infants  RSV induced respiratory failure | neuromuscular disease  uncorrected congenital heart disease  pneumothorax  ventilation >24h  oxygenation index <5  ventilation index <20 |
| Luchetti  2002 | 1 wk to 2 yrs  resp failure due to RSV bronchilitis  PaO_2_/FiO_2_ <150 and PaCO_2_ >40  PIP >35 on CMV at PEEP =0 | head injury with GCS <8  brain death, imminent risk of death or terminal disease  chronic lung disease  preexisting airway disease  congenital heart diseases  neuromuscular diseases |
| Moller  2003 | ARDS Consensus Conference criteria lung injury score at least 2  ventilation between 12 and 120 hrs  age between 44th postconceptual week and 14 years  admission for at least 4 hours  no echocardiographically detectable left heart failure  PaO_2_/FiO_2_ ratio < 100 | NO, HFO, ECMO, liquid ventilation or steroids for ARDS  prostaglandins  chronic lung disease  participation in other clinical trials except treatment protocols and trials for oncological diseases  severe hypoxemia |
| Willson  2005 | age 1 week to 21 years  respiratory failure due to radiographic evidence of bilateral parenchymal lung disease  within 24 hrs of initiation of mechanical ventilation (extended to 48 hrs after initial 50 patients)  oxygenation index > 7 | prematurity  status epilepticus  head injury with GCS < 8  chronic lung disease defined by home O_2_ or diuretic use  brain death  DNR orders  ongoing cardiopulmonary resuscitation  limitation of life support  significant airway disease that may delay extubation  uncorrected congenital heart disease  preexisting myocardial dysfunction  cardiogenic pulmonary edema |

RSV: Respiratory Syncytial Virus

ARDS: Acute Respiratory Distress Syndrome

HFO: High Frequency Oscillatory ventilation

ECMO: Extracorporeal Membrane Oxygenation

PaO_2_ : Arterial oxygen tension

FiO_2_: Fractional inspired oxygen
